# Natural Plant Alkaloid (Emetine) Inhibits HIV-1 Replication by Interfering with Reverse Transcriptase Activity

**DOI:** 10.3390/molecules200611474

**Published:** 2015-06-22

**Authors:** Ana Luiza Chaves Valadão, Celina Monteiro Abreu, Juliana Zanatta Dias, Pablo Arantes, Hugo Verli, Amilcar Tanuri, Renato Santana de Aguiar

**Affiliations:** 1 Laboratório de Virologia Molecular, Departamento de Genética, Instituto de Biologia, Universidade Federal do Rio de Janeiro, Avenida Carlos Chagas Filho 373, Prédio CCS Bloco A, sala 121, 2º andar, CEP: 21941-902, Ilha do Fundão, Rio de Janeiro, Brazil; E-Mails: celydion@gmail.com (C.M.A.); juzdias@gmail.com (J.Z.D.); atanuri@biologia.ufrj.br (A.T.); santana@biologia.ufrj.br (R.S.A.); 2 Grupo de Bioinformática Estrutural, Centro de Biotecnologia, Universidade Federal do Rio Grande do Sul, Avenida Bento Gonçalves 9500, CEP: 91500-970; Porto Alegre, Brazil; E-Mails: pablitoarantes@gmail.com (P.A.); hverli@cbiot.ufrgs.br (H.V.)

**Keywords:** HIV-1, emetine, Reverse Transcriptase, PBMC, resistance

## Abstract

Ipecac alkaloids are secondary metabolites produced in the medicinal plant *Psychotria ipecacuanha*. Emetine is the main alkaloid of ipecac and one of the active compounds in syrup of Ipecac with emetic property. Here we evaluated emetine’s potential as an antiviral agent against Human Immunodeficiency Virus. We performed *in vitro* Reverse Transcriptase (RT) Assay and Natural Endogenous Reverse Transcriptase Activity Assay (NERT) to evaluate HIV RT inhibition. Emetine molecular docking on HIV-1 RT was also analyzed. Phenotypic assays were performed in non-lymphocytic and in Peripheral Blood Mononuclear Cells (PBMC) with HIV-1 wild-type and HIV-harboring RT-resistant mutation to Nucleoside Reverse Transcriptase Inhibitors (M184V). Our results showed that HIV-1 RT was blocked in the presence of emetine in both models: *in vitro* reactions with isolated HIV-1 RT and intravirion, measured by NERT. Emetine revealed a strong potential of inhibiting HIV-1 replication in both cellular models, reaching 80% of reduction in HIV-1 infection, with low cytotoxic effect. Emetine also blocked HIV-1 infection of RT M184V mutant. These results suggest that emetine is able to penetrate in intact HIV particles, and bind and block reverse transcription reaction, suggesting that it can be used as anti-HIV microbicide. Taken together, our findings provide additional pharmacological information on the potential therapeutic effects of emetine.

## 1. Introduction

Human immunodeficiency virus type 1 (HIV-1) is the causative agent of acquired immunodeficiency syndrome (AIDS), which is estimated to affect 35 million people globally, as reported in 2013 [1]. Lymphocytes T CD4+ and monocyte/macrophages are the HIV-1 target cells [2].

As a human retrovirus, once internalized, the HIV RNA genome is reverse-transcribed by Reverse Transcriptase (RT) enzyme to a double-stranded DNA that is further integrated into host DNA [3]. RT activity begins after entry of the virion core into the cytoplasm of the infected cell [4]. HIV-1 RT is a heterodimer formed by two subunits (p66 and p51) and the viral protease is responsible for p51 processing [5]. The p66/p51 heterodimer has two activities: DNA polymerase activity and RNase H activity, which degrades the RNA template associated with the minus-stranded DNA (-ssDNA) [6,7].

RT is an error-prone enzyme without proof-reading activity and with approximately 5–10 nucleotide misincorporations per genome per viral cycle [8]. RT errors together with the high rate of viral replication (1 × 10^10^ virions per day) [9] are associated with high HIV diversity [10]. Highly active antiretroviral therapy (HAART) is the currently available combination therapy for HIV-1 that targets different steps in the virus replication [11]. HAART has considerably improved the quality of life of HIV-infected patients by suppressing viral replication and consequently reducing patients' mortality and rates of hospitalization [12].

RT is a major target for antiretroviral therapy and RT inhibitors include nucleoside analogues (NRTIs) and non-analogues (NNRTIs) [13]. Virus resistance mutations are related to HAART failure and several mutations have been described in the RT gene [14]. K65R mutation is associated with resistance to all NRTIs except zidovudine (AZT); M184V confers resistance to abacavir (ABC), emtricitabine (FTC), and lamivudine (3TC), whereas Y181C mutation confers resistance to all NNRTIs [15]. Therefore, the discovery and characterization of new molecules capable of specific inhibition of HIV remain a high priority [16].

Natural products from plants represent a rich source of structurally novel chemicals that are worth investigating against HIV [17]. Emetine is an alkaloid that possesses a monoterpenoid-tetrahydroisoquinoline skeleton [18]. Emetine and its analogues occur in three plant families including *Alangiaceae*, *Icacinaceae*, and *Rubiaceae*, while the major source of emetine is *Psychotria ipecacuanha* Stokes (Rubiaceae), a native plant from Brazil, also known as *Cephaelis ipecacuanha* A. Rich. [19]. Ipecac root accumulates large amounts of alkaloids. Among them are the two main pharmacologically active components emetine and cephaeline, which represent at least 90% of the alkaloids present in ipecac [20].

A previous study showed that alkaloids extracted from ipecac root were potent inhibitors of HIV RT [21]. However, between the alkaloids tested, emetine exhibited a low *in vitro* potential of inhibition of HIV-1 RT at a concentration of 400 μg∙mL^−1^, but this study was not extended in the context of HIV infection. Emetine and its derivative compounds have long been used in clinic as an emetic, expectorant, spermicide, and anti-amoebic (for dysentery and human fascioliasis), in spite of its variety of toxic manifestations [22,23,24]. Recently, some reports proposed emetine as a candidate for incorporation into chemotherapy regimens for neuroendocrine tumors [25,26], and emetine is also a potent apoptosis inducer in leukemia cells [27]. Emetine has also been mentioned as a candidate for an emergency contraceptive [28].

Here, we extended the analysis of emetine as an anti-HIV agent. Our results demonstrated that emetine blocks HIV-1 infection in primary and established cells in non-cytotoxic levels and its effects are mediated by reverse transcription inhibition. Emetine also blocks HIV-1 infection of a variant strain harboring RT-resistant mutation to NRTIs, suggesting the importance of further studies to evaluate the potential use of this compound as an anti-HIV inhibitor.

## 2. Results and Discussion 

### 2.1. Results

#### 2.1.1. Emetine Impacts on RT Activity

**Figure 1:**
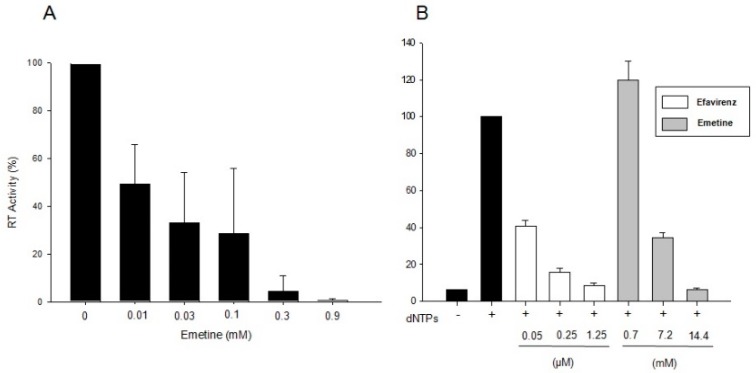
Emetine blocks RT activity. (**A**) RT activity *in vitro* was measured by a colorimetric Reverse Transcriptase Assay. Inhibitory effects of emetine were presented as relative percentage of HIV-1 RT sample that does not contain any inhibitor. We presented the means and errors bars calculated from three independent experiments; (**B**) Emetine blocks intravirion RT activity. HIV viral particles were purified and normalized by ELISA p24. Virus particles treated or not treated with antiretroviral drugs were exposed to dNTPs to stimulate NERT activity. The ssDNA synthesis was evaluated by qPCR 3 h later. The absolute quantification was obtained by comparison with a DNA standard curve (HIV-1 NL4-3 plasmid). All the results are expressed as a percentage related to virus exposed to dNTPs (dNTPs +). At least three replicates of each sample were assayed, and data sets in which the linear correlation coefficient of the standard curve was less than 0.98 were not included for further analysis. dNTPs −: virus not exposed to dNTPs.

Emetine at 0.01 mM blocked 50% of RT activity *in vitro*, suggesting that emetine can directly impact HIV reverse transcription (Figure 1A). We also explored if emetine could block HIV-1 RT activity directly in cell-free virus particles. To address this question, we used the Natural Endogenous Reverse Transcriptase (NERT) assay based on qPCR [29]. The pre-exposure of virus particles to dNTPs stimulate NERT activity (Figure 1B, dNTPs + black bar). However, only basal NERT activity was observed in the absence of dNTPs (Figure 1B, dNTPs − black bar). The presence of emetine (14.4 mM) during NERT reaction reduced up to 90% of the amount of cDNA synthesis compared to the control not exposed to emetine (Figure 1B, gray bars). Efavirenz (1.25 μM) was also able to inhibit endogenous RT activity at the same levels described in previous publications (Figure 1B, white bars). The concentrations applied for NERT assay are expected to be higher since it has been demonstrated that cell-free virions are less permeable to drugs when compared to cell-associated virions [29]. All together, these results show that emetine blocks HIV intravirion RT activity and suggest that this compound could potentially be used as an HIV RT inhibitor. 

#### 2.1.2. Emetine Complexation to RT

**Figure 2:**
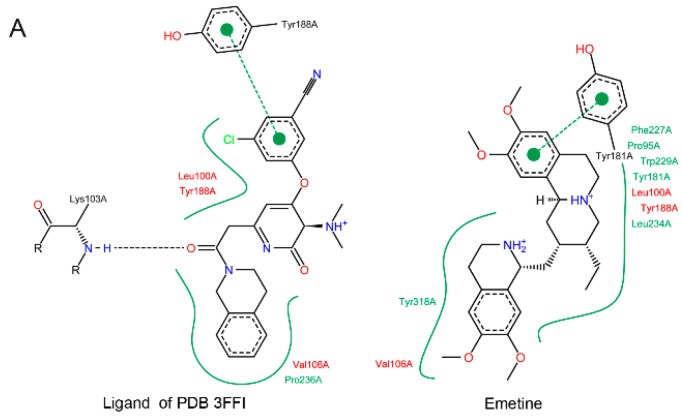

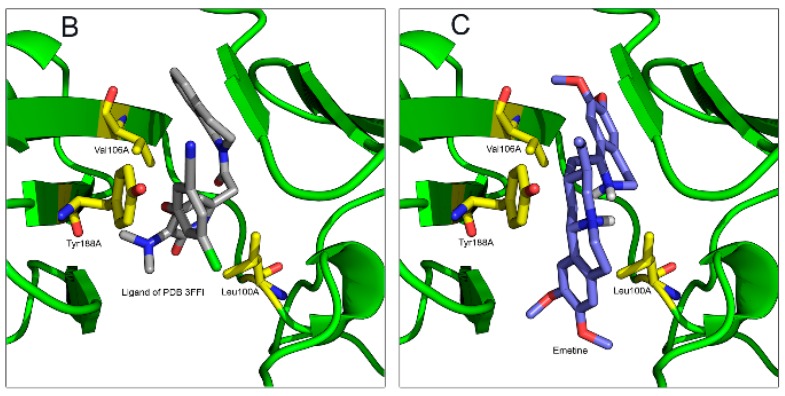
Emetine docking on RT-HIV in comparison with a pyridone analogue. (**A**) Representation of emetine and the pyridone non-nucleosidic analogue binding to RT. The main amino acid residues on compounds binding to RT are presented. Green lines represent hydrophobic pockets, while ref residues indicate interactions observed for both compounds; (**B**) Crystallographic complex between emetine pyridone analogue and RT, and (**C**) RT-emetine complex derived from docking calculations. Common amino acid residues for both complexes are indicated as yellow sticks.

Searching for structural evidence related to the blocking effect of emetine on RT activity, a search on Protein Data Bank was done for potential analogues previously complexed to RT. Accordingly, a pyridone non-nucleosidic inhibitor has been identified (Figure 2A) and employed as a reference for the docking calculations (the same site is shared by other RT inhibitors, such as 3TC). Centered on this analogue binding site, emetine was submitted to docking calculations, including compound flexibility, and the obtained complex was compared to the crystallographic data of its pyridone analogue (Figure 2A), suggesting a similar binding pattern between the two compounds (Figure 2B,C), equivalent amino acid residues, and similar characteristics of the interactions—that is, a predominance of hydrophobic interactions.

#### 2.1.3. Emetine Blocks HIV Infectivity in GHOST Cells

GHOST cells infected with NL4-3-Luc virus allow us to evaluate the effects of emetine in the early stages of HIV infection through luciferase reporter gene activity. For this reason, we checked the virus infectivity of HIV and viruses harboring the RT resistance mutation M184V 48 h post-infection.

**Figure 3:**
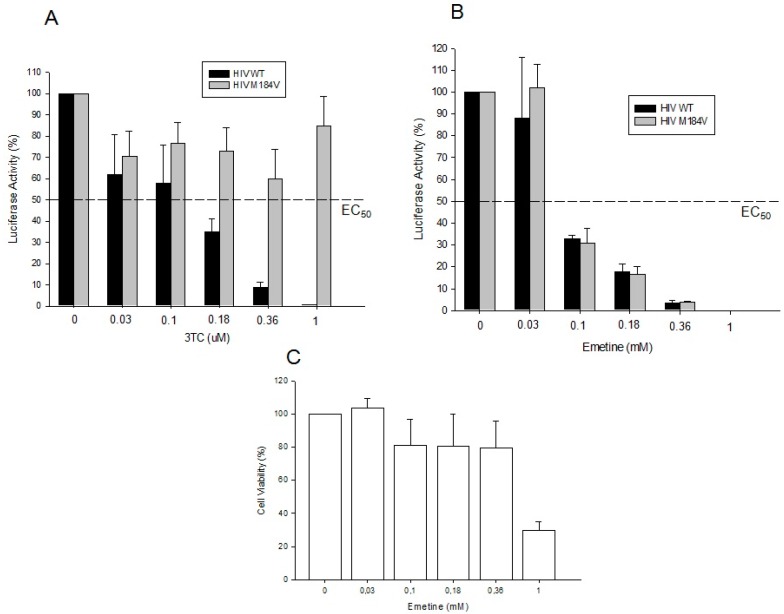
Emetine inhibits HIV wild type and HIV M184V replication in GHOST Cells. (**A**) Resistance profile of HIV WT and HIV M184V in GHOST cells in the presence of 3TC; (**B**) GHOST cells were infected with HIV WT and HIV M184V in the presence of emetine. The HIV infectivity was evaluated by luciferase activity 48 h post-infection from the cell lysates; (**C**) Cytotoxicity effects of emetine were evaluated using fluorescent live/dead staining. All the results are shown in percentage relative to positive controls corresponding to cells not exposed to emetine. Here we present the means and standard deviations of three independent experiments with internal triplicates. The EC_50_ representative value is shown in the graphic.

A control experiment was performed with antiretroviral 3TC in order to guarantee HIV M184V resistance profile (Figure 3A). Emetine blocked HIV infection up to 90% compared with cells without treatment (Figure 3B). Emetine at 0.36 mM blocked 90% of HIV infection with low cytotoxic effects (Figure 3B,C). The same profile of HIV inhibition was observed with HIV M184V virus, meaning that emetine is able to block the replication of HIV-resistant virus. These results suggest that emetine blocks the early stages of HIV infection with 0.1 mM EC_50_ and a therapeutical index of 10.

#### 2.1.4. Emetine Inhibits HIV Infection in Primary Cells

**Figure 4:**
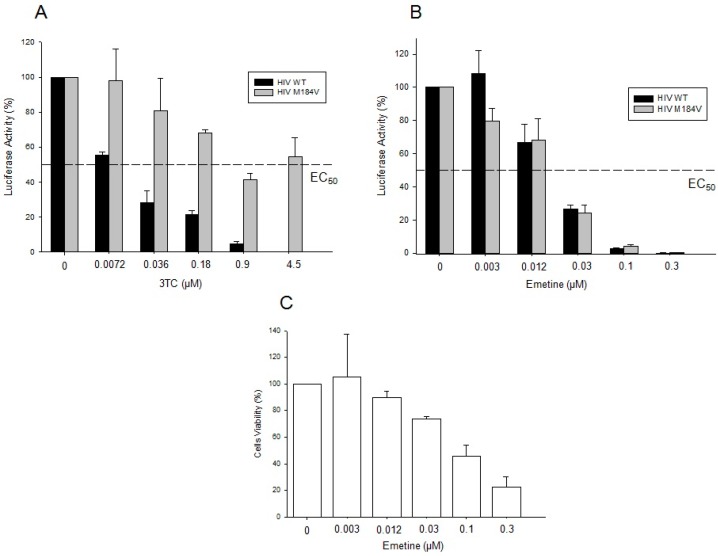
Emetine inhibits HIV wild type and HIV M184V infectivity in PBMCs. Peripheral blood mononuclear cells were isolated from HIV-negative blood donors and infected with HIV WT or HIV M184V and seeded in the presence of an antiretroviral. Viruses' infectivity were evaluated through luciferase activity from PBMCs lysates 96 h post-infection. (**A**) Resistance profile of HIV WT and HIV M184V in PBMCs in the presence of 3TC; (**B**) Phenotypic assays of HIV viruses (WT and M181V) with emetine; (**C**) Cytotoxicity effects of emetine in PBMCs. Peripheral blood mononuclear cells were incubated with increasing concentrations of emetine, followed by propidium iodide staining and flow cytometry analysis. All the results represent the means calculated from three independent experiments with triplicates and all the results are expressed as percentage of untreated cells. The EC_50_ representative value is shown in the graphics.

Next, we evaluated the inhibitory potential of emetine against HIVwild type and RT-resistant virus M184V in PBMCs. The 3TC EC_50_ calculated in PBMCs infected with HIV wild-type virus was 0.0072 µM compared with 4.5 µM for HIV-M184V (Figure 4A). Emetine was also able to block HIV infection in PBMCs with the same profile of GHOST cells. Our results showed that emetine at 0.03 µM reduced up to 80% of the infection of both,HIV wild-type and M184V viruses (Figure 4B) with lower cytotoxic effects (Figure 4C). Finally, those results suggest that emetine is not cytotoxic to human primary lymphocytes and can block NRTI HIV-resistant viruses at micromolar concentrations.

### 2.2. Discussion

Here, we evaluated the inhibitory effect of emetine on HIV-1 replication. Emetine was previously described as inhibiting 10%–20% of RT activity *in vitro* at 400 µg∙mL^−1^ (0.72 µM) [21]. Our data expanded the previous work [21] and we observed that at 0.01 mM of emetine the reduction in HIV RT activity *in vitro* was up to 50%. This concentration is 10 times higher than the one used by Tan and cols [21], and we reached the complete abolishment in RT activity at 0.9 mM of emetine.

NERT assay is based in the principle that HIV virions are biochemically active particles and reverse transcription activity can be stimulated incubating the cell free virus with dNTPs at higher concentrations [30]. Following this idea, emetine could be also captured by the amphipathic domains in the C terminus of the transmembrane glycoprotein (gp41) that accounts for the natural permeability of the HIV-1 envelope to dNTPs [31].

We observed 90% inhibition on intravirion RT activity at 14.4 mM of emetine in cell-free virions. Our NERT inhibition results suggested that emetine penetrates the intact HIV particle and disable its reverse transcription mechanism even before virus infection. These results are very promising, suggesting that emetine could be potentially used as a microbicide anti-HIV-1. Without an effective vaccine, increasing focus is being paid to the development of topical microbicides to prevent the sexual transmission of HIV. Microbicides are topical formulations designed to block HIV-1 infection when applied vaginally (and possibly rectally) before intercourse [32]. To be successful, such agents should: act directly on the virion, act at replication steps prior to the integration of viral DNA into the infected host cell genome, be absorbed by uninfected cells, and finally, be effective at non-cytotoxic concentrations [29]. In fact, some RT inhibitors, such as Nevirapine, Loviride, Tenofovir, and UC781 are now being evaluated as microbicides, and some of them have already been tested in Phase III clinical trials [33,34,35,36].

According to the molecular docking of emetine complexation to the RT structure, we observed an interaction with RT amino acid residues similar to a previously characterized non-nucleosidic analogue (PDB ID 3FFI), suggesting a similar binding pattern of both molecules, with a predominance of hydrophobic interactions.

We expanded our results to non-lymphocytic (GHOST cells) and PBMCs. The result of 80% of HIV inhibition in GHOST cells at 0.1 mM of emetine suggests that this drug targets the early stages of HIV infection. This data seems to correlate with the anti-HIV activity found in RT *in vitro* assays, while higher IC50 values are expected in case of cell based assays, due to the cell permeability issue. For GHOST cells, IC50 values were 10 times higher (0.1 mM) when compared to RT *in vitro* assays (0.01 mM). However, our results for PBMCs showed that emetine at lower concentrations than previously described for *in vitro* inhibition (0.03 µM) was able to block 70%–80% of HIV infection in PBMCs, and this can be due to different cell permeability and incubation time. For PBMCs phenotypic assay, cells were cultivated during four days in the presence of emetine, which can explain the lower IC50 values found in order to avoid cell toxicity.

Emetine EC_50_ value was an intermediate between 0.012 and 0.03 µM in PBMCs, which is very acceptable if compared to other EC_50_ RT inhibitors such as Nevirapine and Zidovudine, which have the EC_50_ values of 0.051 µM and 0.006 µM, respectively, in lymphocytes [37,38]. This low EC_50_ value represents the potency of emetine compared to well-established RT inhibitors. All those results were validated by the non-cytotoxic effects of emetine in those concentrations in the tested cells. Cells were exposed to emetine treatment for up to four days with a 70% profile of cellular viability, suggesting that it is reasonable to consider the use of emetine in clinical protocols. In fact, emetine has been employed as an approved drug in clinical use to treat amoebiasis, with a daily dose of 65 mg over 10 days with an 88% chance of cure [39].

Emetine exhibits a broad spectrum of biological activities, which make it an interesting tool to molecular biologists and pharmacologists. Recently, two reports showed the antiviral property of emetine against poxvirus and dengue virus [40,41]. Emetine at 0.1 µM abolished 99% of vaccinia virus yield and 33% of dengue 2 infection, which is in agreement with our data of 90% of HIV inhibition in PBMCs.

We also evaluated the capability of emetine to inhibit the replication of an HIV-resistant NRTI carrying the M184V mutation. This mutation is responsible for high-level resistance to 3TC and low-level resistance to almost all the NRTIs drugs [42,43]. In contrast with 3TC, emetine was able to block HIV wild-type and M184V virus in GHOST cells and PBMCs, and these results are promising, pointing at a positive use of emetine in the treatment of multidrug-resistant patients. In some countries, the emergence of virus resistance has reached 10%–15% of HIV patients and the development of new antiretroviral drugs that are able to block resistant viruses is essential to control HIV infection [44].

Based on the ability of blocking initial infection, another possible use of emetine in prevention is pre-exposure prophylaxis (PrEP), where emetine could be taken by an HIV-seronegative individual before potential HIV exposure, in order to reduce the risk of HIV infection [45]. PrEP studies have demonstrated HIV-1 protection efficacies ranging from 39% to 73%, but it is likely that the key to efficacy in such trials is adherence to the prophylactic regimen [46,47,48,49,50]. The majority of these studies use a combination of Tenofovir (TNF) and/or Emtricitabine (FTC), due to their desirable characteristics for use as PrEP drugs. However, concern remains over the potential use of these agents in PrEP, regarding the emergence of resistance mutations in the RT gene, which could abolish future treatments with NNRTIs in those patients who have developed resistance to PrEP [51]. Although the resistance to TNF and FTC has been rare and was primarily observed during PrEP initiation in those with acute HIV infection [51], an advantage of taking emetine in PrEP studies lies in the fact that it was effective against HIV M184V mutant virus and this characteristic could overcome the issue of resistance once M184V is a signature mutation for 3TC and FTC [42,52].

Drugs such as emetine, cycloheximide, and dehydroemetine are able to stack mRNA in polysomes and consequently inhibit general translation. In cardiac myocytes, 50% protein synthesis inhibition occurs with 5 × 10^−7^ M emetine (MW 553/mole), which translates to 270 ng∙mL^−1^ [53]. At this concentration, some cardiac toxicity is also observed. However, we already observed a 10-fold reduced infectivity of HIV below this dose (160 ng∙mL^−1^). Nevertheless, topical administration of emetine should have lesser effects and/or the milder dehydroemetine could be considered as an addition to future microbicide strategies against the sexual transmission of HIV. Moreover, emetine analogues, such as emetine ditartrate, were tested as pregnancy interceptive agents in three rodent species, and had an acceptable contraceptive effect by oral routes and vaginal routes in these species [28]. Future studies should address the potential long-term toxicity associated with emetine use, and test if this drug is absorbed into the systemic circulation to any significant extent, and test for the possible emergence of drug-resistant HIV strains.

## 3. Experimental Section

### 3.1. Cell Culture and Reagents

GHOST R3/X4/R5 cells were obtained from the NIH AIDS Research and Reference Reagent Program [52] and the human embryonic kidney cell line HEK-293T was purchased from ATCC. Both cells were cultured in Dulbecco’s modified Eagle’s medium, 10% fetal bovine serum, 100 μg∙mL^−1^ penicillin/streptomycin, and 2 mM glutamine at 37 °C/5% CO_2_. GHOST cells had been engineered to stably express CD4 and the chemokine receptors CCR3, CXCR4, and CCR5 [54] and the expression of these chemokine receptors was maintained by adding Hygromycin (100 μg∙mL^−1^), Geneticin (500 μg∙mL^−1^), and Puromycin (1 μg∙mL^−1^) to the culture medium.

Peripheral blood mononuclear cells (PBMCs) were separated by Ficoll density gradient from buffy coat preparations of healthy blood donors (HIV-negative). PBMCs were grown in RPMI medium, supplemented with 10% human serum and 100 μg∙mL^−1^ penicillin/streptomycin for 48 h, and followed by activation with 100 U IL-2 and 5 μg∙mL^−1^ PHA for five days for HIV-1 infection. Emetine dihydrochloride hydrate (Sigma) was diluted in water and the NRTI 2′,3′-dideoxy-3′-thiacytidine (3TC) was obtained from the NIH AIDS Research and Reference Reagent Program and re-suspended in DMSO (Merck). All the working solutions were prepared by serial dilutions of the stocks in culture medium immediately before use (the final concentration of water or DMSO in working solutions was less than 1%).

### 3.2. Plasmids

HIV-1 proviral clone pNL4-3-Luc has been described previously [55]. The NRTI-resistant mutation M184V was introduced in the RT gene of HIV luciferase proviral clone by the QuikChange Site-Directed Mutagenesis kit (Strategene). The mutagenesis protocol followed the recommendations of the manufacturer and the sequences of the mutagenic primers are: 5′-TATCTATCAATACGTGGATGATTTGTAT-3′(F) and 5′-ATACAAATCATCCACGTATTGATAGATA-3′(R). The construct was sequenced and checked by digestion to confirm the presence of the desired mutation and its integrity.

### 3.3. Viral Production

HIV-1 virus stocks were produced in HEK-293T cells by transfection using FuGENE HD (Promega) with pNL4-3-Luc (HIV) or pNL4-3-Luc M184V (HIV-M184V), following the recommendations of the manufacturer. Viruses were harvested from culture supernatants 48 h post-transfections, and filtered through a 0.22 μM pore-size filter. The viruses' production was evaluated in cell-culture supernatants by ELISA kit against p24 antigen, according to the instructions of the manufacturer (ZeptoMetrix). Virus aliquots for the infection assays were stored at −80 °C. For the NERT assay, viruses were maintained in MT-4 lymphocytes through spinoculation infections (1.200 × g for 2 h) [56]. Virus aliquots were stored at 4 °C for no longer than four weeks, in order to preserve endogenous RT activity. Viruses titrations were performed in MT-4 cells infected with ten-fold virus serial dilutions, and infectivity levels of each virus dilution were evaluated by luciferase measurement, according to the manufacturer’s instructions (Luciferase Assay Kit, Promega).

### 3.4. Infectivity and Phenotypic Assay

GHOST cells were infected with 15 ng of p24 antigen of HIV or HIV M184V in the presence of increasing concentrations of emetine, during an adsorption period of 16 h at 37 °C/5% CO_2_. Cells were washed and fresh medium containing serial dilutions of emetine were added. HIV infectivity was checked 48 h post-infection by luciferase activity in the cell lysates (Luciferase assay kit, Promega). Activated PBMCs (3 × 10^6^ cells) were infected by spinoculation with HIV or HIV M184V at 10^5^ relative luciferase units [57]. Infected cells were seeded in triplicates, in six well-plates (10^6^/well), in RPMI medium containing 100 U IL-2 and 5 μg∙mL^−1^ PHA. Increasing concentrations of Lamivudine (3TC) and emetine were added to evaluate the virus resistance phenotype. Cells were incubated at 37 °C in 5% CO_2_ and 96 h post-infection, cells were lysed and luciferase activity was performed as described above**.** Results are presented as means of three independent experiments with the standard deviations associated. The EC_50_ values in the graphics were calculated with SigmaPlot software, version 9.0.

### 3.5. Cell Viability

To evaluate cytotoxic effects, 10^4^ GHOST cells were seeded in the presence of increasing concentrations of emetine in 96 well-plates. Cell viability was checked at the same time as the point of HIV infections using CellTiter-Blue reagent (Promega). PBMCs viability was accessed by propidium iodide (PI) staining and flow cytometry analysis. Briefly, 2 × 10^6^ activated PBMCs/mL were incubated in six well-plates with increasing concentrations of emetine for four days. Cells were washed in PBS buffer and incubated with 5 μg of PI (Invitrogen) for five min in the dark. The cells' viability was analyzed with a FACSCalibur flow cytometry (BD Biosciences) and the number of viable cells was compared with the control cells not treated with emetine.

### 3.6. In Vitro HIV-1 RT Activity

The inhibitory effect of emetine on RT polymerase activity was assessed by an *in vitro* colorimetric assay (Roche) according to manufacturer’s instructions. The inhibitory activity of emetine was calculated as percent inhibition as compared to a HIV-1 RT sample (2 ng∙mL^−1^) without an inhibitor.

### 3.7. Natural Endogenous Reverse Transcriptase Assay

Natural Endogenous Reverse Transcriptase (NERT) activity has been described elsewhere [29]. Briefly, aliquots with the same amount of purified virus particles, by ultracentrifugation, were incubated with increasing concentrations of emetine or Efavirenz, in a final volume of 50 μL of RPMI culture medium without FBS, for 2 h/37 °C. After this period, virus aliquots were incubated with 20 U of DNase I (Invitrogen) and 10 mM of MgCl_2_ for 1 h/37 °C in a final reaction volume of 25 μL. To stimulate endogenous RT activity, dNTPs mix (2.5 mM) were further added to each tube (except to the negative controls) and the reaction proceeded for 3 h/37 °C. Enzymatic activity was terminated by the addition of 22 μL of a stop solution (10 mM Tris-HCl, pH7.4, 10 mM EDTA, 20 mg∙mL^−1^ of sheared salmon sperm DNA, 50 mg∙mL^−1^ of proteinase K) and heated at 95 °C/10 min to disrupt virus particles. Reaction mixtures were stored at −70 °C until they were quantified by qPCR. The amounts of newly synthesized double-stranded strong stop DNA (ssDNA) were measured by qPCR, using the following HIV-1-specific oligonucleotides and probe: SSF1 (5′-GCTAACTAGGGAACCCACTGCTT-3′), SSR1 (5′-CAACAGAC GGGCACACACTACT-3′), and ssDNA probe (5′FAM-AGCCTCAATAAAGCTTGCCTTGAGTGCTTC-BHQ1-3′). Reaction mixtures were as follows: 1 μL Taqman Universal PCR mixture (Applied Biosystems, Foster City, CA, USA), 0.25 pmol of each primer, 0.05 pmol ssDNA probe, and 4 μL of NERT stopped reaction mixture in a final volume of 25 μL. Amplifications were performed in the 7500 Sequence Detection System (Applied Biosystems). We used 50 °C/2 min, 95 °C/10 min, and 50 cycles at 95 °C/15 s and 60 °C/min as cycle conditions. Serial dilutions of HIV proviral clone were performed in NERT stop solution and used as qPCR standard curves to absolute quantification (10^7^–10 copies/μL).

### 3.8. Emetine Docking on HIV-1 RT

The construction of emetine was performed with Molden [58], and the *ab initio* calculations were performed using GAMESS [59]. CHELPG atomic charges were employed for the ligand, obtained from the HF/6-31G** level. Docking procedures were realized with Autodock, version 4.2 [60]. The RT-HIV crystal structure complexed with an analogue of emetine was used (PDB 3FFI), and the docking was centered in its binding site. The original ligand and water molecules were removed prior to docking procedures and only polar hydrogens were added. The Lamarckian Genetic Algorithm (LGA) [61] was used to explore the space of the binding site, centered on the emetine site on RT-HIV. For each run, a maximum number of energy evaluations was set to 25,000,000, and a maximum number of 27,000 LGA operations were generated on populations of 10 individuals. A total of 100 runs were performed, and a population of 1000 individuals was employed. Crossover, mutation, and elitism were set to 0.80, 0.02, and 1, respectively. All rotatable dihedrals were treated as flexible during the calculations. The orientation of emetine at the binding site was selected from the lowest energy docked conformation generated by Autodock. The 2D images were generated with the PoseView server [62], and 3D images were performed using Pymol program [63].

## 4. Conclusions

Drug combination regimens for AIDS treatment are designed to prevent the emergence or replication of multidrug-resistant HIV-1 strains and our findings of emetine inhibition of NRTIs-resistant virus (M184V) in human primary cells provide additional information on the potential therapeutic use of emetine. We also consider that emetine can be used as a starting point for chemical modifications in order to improve potency and selectivity for HIV RT.
